# Supplementary material for: Effects of Combined Use of Olive Mill Waste Compost and Sprinkler Irrigation on GHG Emissions and Net Ecosystem Carbon Budget under Different Tillage Systems
Source: Plants (Basel). 2022 Dec 9;11(24):3454. doi: 10.3390/plants11243454 (PMC9784570; doi:10.3390/plants11243454)
Supplement: Supplementary file 1 [file plants-11-03454-s001.zip › plants-2075477-supplementary.pdf]

SUPPLEMENTARY TABLE

Table S1. Effects of different management systems on soil physicochemical properties (0–20 cm depth).

	TOC ($g\ kg^{-1}$)	WSOC ($mg\ kg^{-1}$)	FA ($g\ kg^{-1}$)	HA ($g\ kg^{-1}$)	HI	EC ($dS\ m^{-1}$)	pH	N ($g\ kg^{-1}$)	P ($mg\ kg^{-1}$)
**2015**									
DS	10.2 aA	210 bA	1.10 bC	1.62 bB	15.9 cB	2.14 aA	5.77 bA	0.725 aA	34.5 aB
DSC	21.3 cC	448 cA	1.29 cB	2.35 dB	11.0 aB	3.69 bAB	6.58 dB	1.82 bA	38.3 bB
T	10.7 aA	137 aA	0.965 aA	1.34 aB	12.5 abB	3.74 bB	5.64 aB	0.805 aA	34.9 aA
TC	15.6 bA	497 dB	1.29 cC	2.02 cB	12.9 bB	4.19 cB	5.99 cA	1.53 bA	35.7 aA
**2016**									
DS	9.99 aA	309 aB	0.810 aA	1.14 aA	11.4 cA	2.49 cA	5.86 bB	1.52 aB	37.0 aC
DSC	16.5 cA	533 bA	0.929 aA	1.35 abA	8.16 aA	3.63 dB	6.42 cA	2.37 cA	40.8 abB
T	11.3 bA	335 aB	0.920 aA	1.17 aA	10.3 bcA	2.21 aA	5.41 aA	1.59 aB	40.9 abC
TC	17.4 dB	519 bB	0.761 aA	1.59 bA	9.14 abA	2.40 bA	6.56 dC	2.15 bB	43.4 bB
**2017**									
DS	15.1 bB	316 aC	0.946 aB	1.44 aAB	9.53 aA	3.96 bB	6.08 bC	1.81 bC	30.1 aA
DSC	19.1 dB	498 bA	1.24 cB	2.24 bB	11.7 bC	3.44 aA	6.61 dB	2.38 dA	34.6 bA
T	13.1 aB	336 aB	1.48 dB	1.55 aC	11.9 bB	4.62 cC	5.61 aB	1.67 aB	36.9 cB
TC	18.2 cB	395 aA	1.13 bB	2.40 bC	13.2 cB	3.73 abB	6.35 cB	2.24 cB	36.8 cA
Y	123***	9.76**	108***	92.2***	67.2***	156***	39.8***	75.1***	39.2***
T	1020***	32.5***	17.3**	64.2***	9.92**	31.8***	580***	53.2***	17.7**
YxT	60.7***	3.89*	22.2***	10.2***	19.4***	55.3***	49.6***	1.36NS	4.04*

Rice cultivated by, DS: direct seeding; DSC: direct seeding compost; T: Tillage; TC: Tillage compost. TOC: Total Organic Carbon; WSOC: Water Soluble Organic Carbon; FA: Fulvic Acid; HA: Humic Acid; HI: Humification index; EC: Electrical Conductivity; N: Total Nitrogen; P: Available Phosphorus. ANOVA factors are Y: Year; T: Treatment; Y x T: Interaction Year * Treatment. F-values indicate the significance levels * $p < 0.05$; ** $p < 0.01$; *** $p < 0.001$, respectively, and NS: not significant. Different letters indicate differences ($p < 0.05$) between treatments in the same year (lower case letters) and between years within the same treatment (upper case letters).

Table S2. Effects of different management systems on soil enzyme activities (0–10 cm depth).

	DH (µg INTF g^{-1} h^{-1})	GL (µmol pNP g^{-1} h^{-1})	UR (µg NH_4^+ g^{-1} h^{-1})	PHO (µmol pNP g^{-1} h^{-1})	ARS (µg pNP g^{-1} h^{-1})
**2015**					
DS	0.993 aA	0.210 aA	17.7 aA	1.46 aA	9.96 abA
DSC	1.89 bB	0.980 bA	45.2 bA	1.28 aAB	7.34 aA
T	1.06 aB	0.233 aB	17.7 aA	1.23 aA	7.98 aA
TC	1.35 abA	1.20 bA	26.1 aA	1.30 aA	14.5 bA
**2016**					
DS	0.663 abA	0.157 aA	19.9 abA	1.59 aA	13.1 aA
DSC	1.13 cAB	2.33 bA	28.5 bA	1.67 aB	16.2 aB
T	0.510 aA	0.117 aA	8.00 aA	1.56 aB	14.0 aA
TC	0.877 abA	1.12 aA	20.6 abA	1.57 aA	15.0 aA
**2017**					
DS	0.713 abA	0.077 aA	13.9 aA	1.41 aA	9.16 aA
DSC	1.09 bA	1.48 bA	27.7 bA	1.20 aA	20.4 bC
T	0.333 aA	0.093 aA	9.23 aA	1.40 aAB	10.4 aA
TC	0.827 bA	0.890 abA	25.9 bA	1.41 aA	19.4 bA
Y	23.2***	2.76NS	6.33**	8.24**	8.49**
T	5.11*	13.29**	12.9**	1.23NS	8.94**
YxT	0.882NS	3.32*	2.02NS	0.749NS	3.22*

Rice cultivated by, DS: direct seeding; DSC: direct seeding compost; T: Tillage, and TC: Tillage compost. DH: Dehydrogenase Activity; GL: B-Glucosidase Activity; UR: Urease Activity; PHO: Phosphatase Activity; ARS: Arysulfatase Activity. ANOVA factors are Y: Year; T: Treatment; Y x T: Interaction Year * Treatment. F-values indicate the significance levels * $p < 0.05$; ** $p < 0.01$; *** $p < 0.001$, respectively, and NS: not significant. Different letters indicate differences ($p < 0.05$) between treatments in the same year (lower case letters) and between years within the same treatment (upper case letters).
